# Sex Chromosomes and Internal Telomeric Sequences in *Dormitator latifrons* (Richardson 1844) (Eleotridae: Eleotrinae): An Insight into Their Origin in the Genus

**DOI:** 10.3390/genes11060659

**Published:** 2020-06-17

**Authors:** Fabilene Gomes Paim, Mauro Nirchio, Claudio Oliveira, Anna Rita Rossi

**Affiliations:** 1 Departamento de Biologia Estrutural e Funcional, Instituto de Biociências Universidade Estadual Paulista-UNESP, Botucatu 18618-689, São Paulo, Brazil; fabillene@yahoo.com.br (F.G.P.); claudio@ibb.unesp.br (C.O.); 2 Universidad Técnica de Machala, Av. Panamericana km 5½, Vía Pasaje, Machala 070151, El Oro, Ecuador; mauro.nirchio@gmail.com or; 3 Escuela de Ciencias Aplicadas del Mar, Núcleo de Nueva Esparta, Universidad de Oriente, Porlamar 6301, Isla de Margarita, Venezuela; 4 Dipartimento di Biologia e Biotecnologie “C. Darwin”, Sapienza-Università di Roma, Via Alfonso Borelli 50, 00161 Rome, Italy

**Keywords:** XX/XY sex system, FISH, rDNA, (TTAGG)n, ITS, Pacific fat sleeper

## Abstract

The freshwater fish species *Dormitator latifrons*, commonly named the Pacific fat sleeper, is an important food resource in CentralSouth America, yet almost no genetic information on it is available. A cytogenetic analysis of this species was undertaken by standard and molecular techniques (chromosomal mapping of 18S rDNA, 5S rDNA, and telomeric repeats), aiming to describe the karyotype features, verify the presence of sex chromosomes described in congeneric species, and make inferences on chromosome evolution in the genus. The karyotype (2n = 46) is mainly composed of metacentric and submetacentic chromosomes, with nucleolar organizer regions (NORs) localized on the short arms of submetacentric pair 10. The presence of XX/XY sex chromosomes was observed, with the X chromosome carrying the 5S rDNA sequences. These heterochromosomes likely appeared before 1 million years ago, since they are shared with another derived *Dormitator* species (*Dormitator maculatus*) distributed in the Western Atlantic. Telomeric repeats hybridize to the terminal portions of almost all chromosomes; additional interstitial sites are present in the centromeric region, suggesting pericentromeric inversions as the main rearrangement mechanisms that has driven karyotypic evolution in the genus. The data provided here contribute to improving the cytogenetics knowledge of *D. latifrons*, offering basic information that could be useful in aquaculture farming of this neotropical fish.

## 1. Introduction

Freshwater habitats, although covering a small proportion of the Earth’s surface (about 1%) [1], host rich biodiversity, largely represented by freshwater fishes [2]—about 40% of global fish diversity [3]. The Neotropical Region shows remarkable diversification of species and hosts more than three-quarters of the world’s fish functional diversity [4]. For the continental Neotropical Region 20 orders, 69 families and 5160 freshwater fishes have been described, accounting for about 40% of worldwide freshwater biodiversity [5]. Among the thousands of native fish, many play an important role in the economies in this geographic area as a source of animal protein for riparian communities or, on a more industrial scale, for fish farming, commercial fishing, aquarism, and recreational sports [6].

Research on fish systematics and evolution has expanded over time, including, in recent decades, molecular taxonomy, developmental biology, and new tools like 3D imaging [2]. Cytogenetic analyses join these new tools, providing new evidence of the existence of cryptic species and evolutionary divergence ([7] and references therein) and giving rise to a prosperous field focused on neotropical species [8,9].

Eleotridae fishes are distributed circumglobally, but mostly in tropical and subtropical areas. This family includes 171 valid species grouped into three subfamilies: Butinae, Eleotrinae, and Milyeringinae [10]. The species-rich subfamily Eleotrinae contains 19 genera [11], one of which, *Dormitator* Gill, 1861 includes amphidromous freshwater fishes distributed in the tropical and subtropical coastal waters of the Atlantic and the American Pacific coasts. Four species are known from America, three in the Western Atlantic (*Dormitator cubanus* Ginsburg, 1953, *Dormitator levis* (Eigenmann, 1918), *Dormitator maculatus* (Bloch, 1792)), and one in the Eastern Pacific (*Dormitator latifrons* (Richardson, 1844)); a fifth one (*Dormitator lebretonis* (Steindachner, 1870)) is endemic to the West African coastline from Senegal to Namibia [12]. The distribution of *Dormitator* has been interpreted as the result of vicariance that followed the break-up of Gondwana, with transisthmian differentiation of two main lineages: one composed of the species sampled in the Western Atlantic basin, and the other represented by the species from the Pacific basin. Although two morphological subspecies have been proposed in this area—*Dormitator latifrons mexicanus* Ginsburg 1953, restricted to the Pacific coast of Mexico, and *Dormitator latifrons latifrons*, present in South America—molecular data showed the absence of phylogeographic structure in the Pacific area, that is, the data did not support morphological differentiation ([13] and references herein).

*D. latifrons*, commonly known as the Pacific fat sleeper is distributed from southern California, USA and the Gulf of California, Mexico to Ecuador, including the Galapagos Islands [14]. This species represents a food source [15,16], and in Ecuador it is of great economic importance for coastal communities, where it is cultivated in an artisanal way for consumption and export [17]; moreover, in Mexico and Nicaragua, there is interest in developing its production by aquaculture [18]. Indeed, *D. latifrons* due to its high-quality flesh, fast growth rate and low protein requirements has been identified as a possible candidate to replace tilapia species [19,20] that are cultivated in Ecuador in order to mitigate the impact of these invasive cichlids on Ecuadorian biodiversity [21]. Thus, any effort made to improve basic research on the species is of great importance for the development of cultivation programs.

The inclusion of cytogenetic screening in fish biology and aquaculture, may be very informative and useful in studies on systematic and taxonomic relationships, chromosomal polymorphism, interspecific hybridization, early embryogenesis, chromosome set manipulations, and sex determination mechanisms [22,23,24]. To date, cytogenetic data on Eleotridae scantly describe the diploid number for 11 species (Table 1), barely representing 6.4% of the species ascribed to the family. The karyotype of *D. latifrons* was already described [25,26,27,28,29,30,31,32,33,34], but the information provided is limited to the diploid number and the karyotype composition. Data from C-bands and active nucleolus organizer regions (Ag-NORs) are limited to other two species of the family, *D. maculatus* and *Eleotris pisoni* [28,29]. In the former species, sex chromosomes were described in a population from Brazil.

Comparisons of sex determination systems between closely related fish species can provide important insights into the evolution of sex chromosomes [35,36]. Thus, in this study a cytogenetic survey of *D. latrifons* was undertaken, aimed to verify whether sex chromosomes could be identified in this species and to clarify whether these chromosomes represent a common feature in the genus, i.e., whether they appeared before the split of the Pacific and Atlantic *Dormitator* lineages [13] or, alternatively, arose recently in the Atlantic lineage. For this purpose, besides standard staining (Giemsa, silver nitrate, C-banding), the use of repetitive sequences (18S rDNA, 5S rDNA, and telomere repeats) mapping was applied. Indeed, ribosomal genes (rDNA) belong to the most investigated [37] and informative [38] repeats in fish, wherein they have extra-coding functions and take part in genome diversification associated with speciation events [38,39]. This approach allows us to highlight eventual chromosome rearrangements that could have occurred during karyotype evolution in the genus and could be associated with sex chromosome differentiation. The data will contribute to improving the knowledge of the biology of *D. latifrons* and Neotropical fish cytogenetics, also providing basic information that could be useful in aquaculture farming of this species.

## 2. Materials and Methods

### 2.1. Specimens Collection and Identification

Fifteen specimens of *D. latifrons* (seven males, eight females) were collected using a seine net in La Tembladera wetland (3°29′13.5″ S 80°00′00.9″ W) and transported to the laboratory in sealed plastic bags with water and a supply of pure oxygen. Sample identification was subsequently performed [40]. Voucher specimens fixed in 10% formalin were deposited in the Ichthyology Collection of the Laboratório de Biologia e Genética de Peixes (LBP) of Universidade Estadual Paulista, Botucatu, São Paulo, Brazil (UNESP) (collection numbers LBP 29080) and Universidad Técnica de Machala (collection numbers UTMACH 0365, 0372, 0373, 0377, 0378, 0381, 0383, 0394, 0395). Procedures were conducted with Institutional Research Project Authorization (PR-GEN-155), following the ethical/anesthesia conduct approved by the Ethics Committee on Animal Experimentation of the Universidad Técnica de Machala (process number UTMACH-CEEA-002-2020-AC).

### 2.2. Cytogenetic Procedures

Twenty-four hours before chromosome preparations, the fishes were injected intramuscularly with a yeast glucose solution [41] for mitosis stimulation. Each fish received an intra-abdominal injection of 0.0125% colchicine (1.0 mL/100 g of body weight) 50 min before being sacrificed by a numbing overdose of benzocaine (250 mg/L) as recommended by the American Veterinary Medical Association [42]. Mitotic chromosomes were obtained following the conventional air-drying method from a kidney cell suspension [43].

For the conventional karyotype, slides were stained for 20 min with 10% Giemsa in phosphate buffer at pH 6.8. Active nucleolus organizer regions (Ag-NORs) and the constitutive heterochromatin distribution were revealed by silver staining [44] and C-banding [45], respectively.

Assays for mapping onto chromosome 5S rDNA, 18S rDNA (ribosomal genes), and telomeric repeats were performed via fluorescence in situ hybridization (FISH) [46]. Probes for 5S rDNA, 18S rDNA, and telomeric repeats were obtained and labeled by PCR using primers and procedures previously reported [7]. Biotin-labeled probes were detected with fluorescein-conjugated avidin (FITC, Sigma-Aldrich, St. Louis, MO, USA) and digoxigenin-labeled probes were visualized using anti-digoxigenin-rhodamine conjugate (Roche Applied Science, Basel, Switzerland). After detection, the chromosomes were counterstained with 4,6-diamidino-2-phenylindole (DAPI) included in the Vectashield mounting medium (Vector Laboratories Ltd.,Peterborough, UK).

Metaphase cells analyzed by conventional techniques were photographed using a Motic B400, equipped with a Moticam 5000C digital camera using Motic Images Plus 2.0 ML software. FISH images were captured using an Olympus BX61 photomicroscope (Olympus Corporation, Ishikawa, Japan) equipped with a DP70 digital camera using Image-Pro Plus 6.0 software (Media Cybernetics, Silver Spring, MD, USA). Images were merged and edited for optimization of brightness and contrast using Photoshop (Adobe Systems, Inc., San Jose, CA, USA) Version 2015.0.0. Chromosomes were arranged in decreasing order and classified according to centromere position [47].

## 3. Results

**Figure 1:**
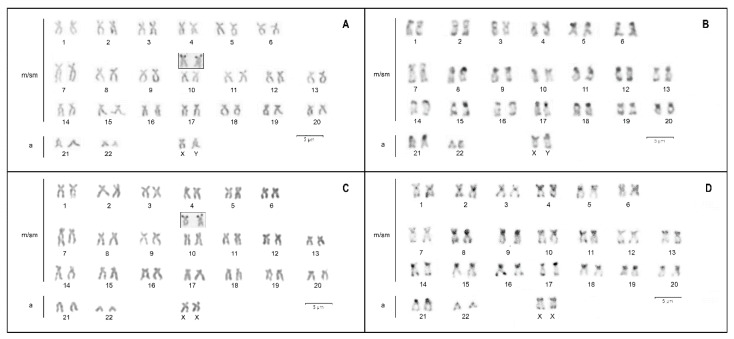
Conventional Giemsa and C-banded karyotypes of male (**A**,**B**) and female (**C**,**D**) *D. latifrons*. m/sm, metacentric/submetacentric, a acrocentric. Heteromorphic sex chromosomes are indicated. Insets of NOR-carrying chromosomes, after silver staining, are shown in (**A**,**C**).

The analysis of 496 mitotic metaphase cells of *D. latifrons* revealed a modal diploid number of 2n = 46 for both males and females (98.7% of cells) but a clear karyotypic difference was observed between sexes. In females, the karyotype consisted of 42 metacentric/submetacentric (m/sm), and four acrocentric (a) chromosomes, whereas males presented 41 m/sm and five a chromosomes (Figure 1A,C).

Impregnation with silver nitrate showed a single pair of Ag-positive NORs terminally located on the short arms of sm pair 10 (Figure 1A,C, inset), in both sexes.

Constitutive heterochromatin appears to be distributed in the pericentromeric and telomeric regions of all chromosomes (Figure 1B,D). C-positive blocks are also recognizable on the short arms of chromosome pair 10 carrying NORs and on those of the X chromosome; in the Y chromosome, besides the short arms, heterochromatin can also be observed in the long arms, close to the pericentromeric area.

**Figure 2:**
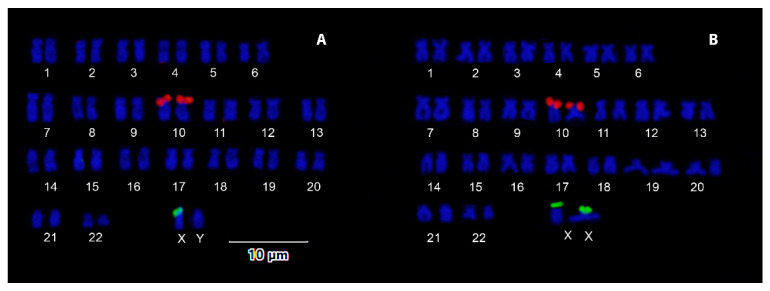
Karyotypes of *D. latifrons* arranged after 5S (green) and 18S (red) rDNA double fluorescence in situ hybridization (FISH): (**A**) male, (**B**) female.

Double FISH assays with ribosomal genes showed that 18S rDNA sequences are localized on a single chromosome pair (likely the same that is Ag positive) in both males and females, whereas 5S rDNA genes have a differential distribution in the two sexes (Figure 2A,B). Indeed, in females, the minor rDNA clusters occur terminally on one of the m/sm chromosome pairs, whereas in males, positive signals are present on a single m/sm chromosome. Differences in the size of the single 5S rDNA signals were not observed between the two sexes. Thus, it is likely that these genes are localized on the X chromosome.

**Figure 3:**
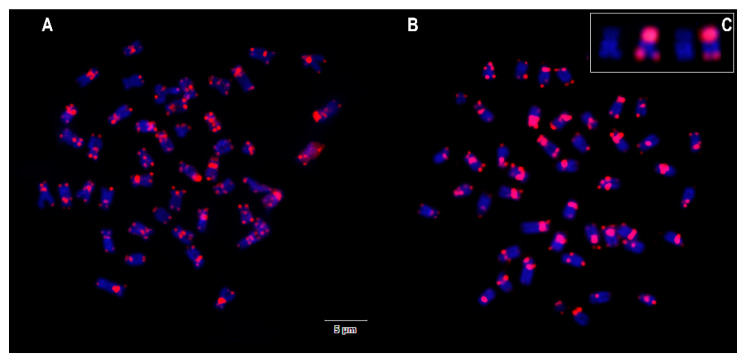
Metaphases of male (**A**) and female (**B**) *D. latifrons* under FISH with telomeric probes and counterstaining by DAPI. Many interstitial positive sites are present in both sexes. Enlargement of samples of the NOR-carrying pair in DAPI (left) and under FISH with telomeric probes, (right) is shown in the inset (**C**).

The sequences of repetitive telomeric DNA hybridized at the terminal portions of almost all chromosomes; additional interstitial telomeric sequence (ITS) signals are highlighted in the centromeric region of most chromosomes. In some chromosomes, both the terminal and the interstitial locations of (TTAGGG)n repeats appear as very big, bright blocks (Figure 3). No differences were observed between males and females. The big positive blocks that cover the entire short arms of a submetacentric pair correspond to secondary constriction, i.e., to NORs that are faintly stained by DAPI (Figure 3C).

## 4. Discussion

Cytogenetic studies performed in 11 species of Eleotridae belonging to the subfamilies Butinae (two species) and Eleotrinae (nine species) showed that in this family the diploid number 2n ranges from 46 to 52, with a predominance of uni-armed chromosomes in the karyotypes (Table 1). In *Dormitator*, previous cytogenetic studies reported karyotypes with 2n = 46 chromosomes, mainly composed of bi-armed chromosomes (chromosome arm number (FN) = 86−90) for both *D. latifrons* and *D. maculatus*. Our data confirm this diploid number for *D. latifrons*, although with a different karyotype composition (Table 1).

In addition, here we report differences in the karyotypes of males and females of *D. latifrons*, that suggest an XX/XY sex chromosome system not reported by previous studies on samples from Mexico [25,26]. As a total absence of phylogeographic structure was detected in *Dormitator* across the Pacific area [13], this difference likely does not reflect geographic differentiation but is instead due to the fact that in previous surveys only Giemsa staining was used.

**Figure 4:**
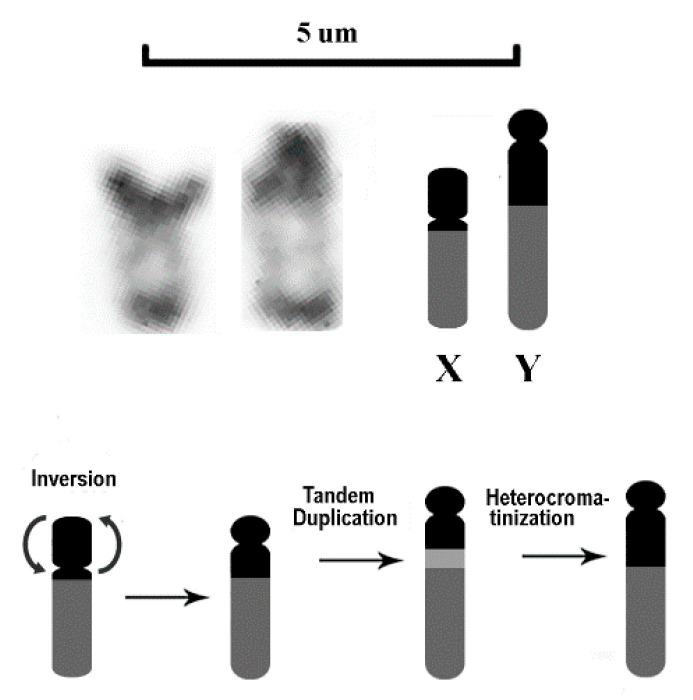
C-banded enlargement and schematic representation of sex chromosomes (above) and the hypothetical mechanism of chromosomal rearrangements that allowed the origin of the, Y chromosome (below).

The presence of cytologically distinguishable sex chromosomes (CDSC) characterizes only a minority of fish, corresponding to less than 1% of Teleosts [48,49] and about 10% of the karyotped fish species [50,51,52,53,54], as in most of them the sex chromosomes are cryptic. CDSC largely appeared independently and at various times in different fish lineages, following distinct patterns of differentiation even in closely related species ([53,55] and references herein); they can be associated to male or female heterogamety, or even to multi-chromosomal sex determination [56]. In both cases, the presence of transposable elements is crucial for the differentiation and evolution of these chromosomes [57]. The emergence of CDSC requires the suppression of homologous recombination by structural rearrangements and repetitive DNA accumulation [53,58,59,60]. In *D. latifrons*, the presence of an additional heterochromatic block on the Y chromosome, besides the one that covers entirely the short arm of the X chromosome, suggests a scenario of structural rearrangement that, independent of the order of occurrence, would include a pericentric inversion of the heterochromatic region of the p arm on one of the X chromosomes, a tandem duplication, and the subsequent accumulation of repetitive DNA in the Y chromosome (Figure 4). These chromosomal rearrangements are associated with a reduction/loss of the 5S rDNA sequences in males (see below). Similar XX/XY sex chromosomes were reported in *D. maculatus* from Brazil [29], and this makes it possible to infer their origin. Indeed, as Pacific and Atlantic *Dormitator* geminate lineages diverged about 1 million years ago following geological and oceanographic changes associated with the formation of the Central American Isthmus [13], the differentiation of these sex chromosomes has an ancient origin and should have occurred before the splitting of the genus between the two ocean basins. In addition, as *D. maculatus* represents the most derived branch of Atlantic *Dormitator*, the presence of sex chromosomes could be expected in the other species of the genus.

In *D. latifrons*, the number and location of major ribosomal genes conform to the most common condition detected in Teleosts, i.e., a single bearing chromosome pair *per* diploid karyotype present or associated to the terminal regions of chromosomes [37]. On the contrary, chromosomal mapping of 5S rDNA on sex chromosomes is not a common condition in fish, but rather an exception [39,61,62] that obviously determines differences in the number of bearing chromosomes between the two sexes. It remains unclear whether minor ribosomal genes are totally absent in the Y chromosome or present in reduced copy number, which does not allow their detection [61]. We might infer, in any case, an unbalance in copy number between male and female exists, and, thus, an unbalance between the copy numbers of 5S and 45S rDNA. In lower vertebrates, including fish, discrepancies between the copy numbers of these genes are tolerated and play a role in the microevolutionary processes, making these repetitive sequences good markers to trace population diversification (see [38] for a review). Meanwhile, recent research demonstrated that in fishes the role of rDNA in maintenance genome integrity can be associated to a change in copy number in response to environmental conditions [63], according to the idea of a “Musical Chair” model for rDNA copy counting [64].

The mapping of telomeric (TTAGGG)n probes in vertebrate chromosomes showed that telomeric motifs could also be located interstitially along chromosome arms (interstitial telomeric sequences, ITSs), in pericentromeric/centromeric regions, or between the centromere and the telomere [65,66]. In fish chromosomes, these non-functional telomeres allow us to better elucidate chromosomal rearrangements in specific taxonomic groups [7,62] or to disentangle the evolutionary relationships of ancient species and wider taxonomic groups [67]. In the genome of *D. latifrons*, many ITSs were detected in pericentromeric regions, and some correspond to heterochromatic ITSs (het-ITSs), i.e., blocks of several hundreds of kilobases within or close to heterochromatic areas [68], that have been identified in different vertebrate species [65]. Among these, we can include the large ITS signals that overlap with NORs; these are the result of the interspersion of the two types of repeated sequences, as observed in many fish species [69,70,71,72]. Heterochromatic ITSs have been proposed to originate by a four-step mechanism that involves several chromosomal rearrangements associated with the amplification and redistribution of (TTAGGG)n repeats [65], thus behaving like satellite DNA. Regardless of their heterochromatic or not, ITSs are considered very unstable microsatellite sequences, able to trigger chromosomal rearrangements [73], and in fish are generally considered as relicts of chromosome fusions [74] or of transposition events [62]. In *D. latifrons* the presence of a large number of ITSs without a reduction in the diploid number suggests chromosome evolution by pericentric inversions, according to what has been observed in other fish species [66,75]. This is congruent with the idea that chromosomal fusions to be stabilized require telomeric loss or inactivation in centromeric position [76], but not in the interstitial position, probably because in this case there is no problem with mitotic or meiotic fuse formation.

## 5. Conclusions

The data here reported provide useful information on the presence of sex chromosomes in *D. latifrons* and suggest an ancient origin for these heterochromosomes (before 1 million years ago), as the same XY system is shared with another *Dormitator* species (*D. maculatus*) present in the Western Atlantic. The presence of many ITSs—regions that are considered hotspots of recombination [74]—suggests the occurrence of many pericentromeric inversions during the chromosomic evolution of this genus. Chromosome localization of telomeric repeated sequences in other *Dormitator* and in other Eleotrinae species is necessary to get a more comprehensive picture on the role of such sequences in the karyotype evolution of this group. In addition, chromosomal mapping of transposable elements [39] could better define the contribution, if any, of these repeated sequences in the process.

Finally, the basic cytogenetics information here provided, like the presence of sex chromosomes, could open a path for studies of genetic manipulation [77,78] or marker-assisted selection [79] in *D. latifrons*, aimed at improving productive characteristics, in order to make this species an alternative to tilapia in the Ecuadorian aquaculture industry. Indeed, the Pacific fat sleeper, due to its high tolerance to variations in salinity and temperature and its low oxygen requirement, may prove useful as aquaculture shifts in the face of climate change [19]. At the same time, the replacement of tilapia in aquaculture could have beneficial effects on Ecuadorian biodiversity [21].

## Figures and Tables

**Table 1:** Cytogenetic data in Eleotridae fishes: diploid chromosome number (2n), number of chromosomes in each morphological category (m, metacentric; sm, submetacentric; st, subtelocentric; a, acrocentric), chromosome arm number (FN).

Species	2n	Karyotype Composition	FN	Sampling Area	References
**Eleotrinae**					
*D. latifrons*	46	44m/sm + 2st/a	90	Mexico	[25]
*D. latifrons*	46	44m/sm + 2st/a	90	Mexico	[26]
*D. latifrons*	46	42m/sm + 4a (♀) 41m/sm + 5a (♂)	88/87	Ecuador	This paper
*D. maculatus*	46	44m/sm + 2st/a	90	Mexico	[26]
*D. maculatus*	46	34m/sm + 12st/a	80	Brazil	[27]
*D. maculatus*	46	40m/sm + 6st/a	86	Brazil	[28]
*D. maculatus*	46	14m + 28sm + 2st + 2a (♀); 13m + 28sm + 3st + 2a (♂)	90	Brazil	[29]
*Eleotris acanthopoma*	46	46st/a	46	Japan	[30]
*Eleotris oxycephala*	46	46a	46	China	[30]
*E. oxycephala*	46	46a	46	China	[30]
*Eleotris picta*	52	52a	52	Mexico	[31]
*E. pisonis*	46	2m/sm + 42st/a	46	Mexico	[31]
*E. pisonis*	46	46a	46	Unknown	[32]
*E. pisonis*	46	46a	46	Brazil	[28]
*Gobiomorus dormitor*	48	6m/sm + 42a	54	Mexico	[27]
*Hypseleotris cyprinoides*	48	48a	48	Japan	[30]
*Mogurnda mogurnda*	46	6sm + 40st/a	52	Australia	[30]
**Butinae**					
*Ophiocara porocephala*	48	48a	48	Thailand	[30]
*Oxyeleotris marmorata*	46	2m + 2sm + 42a	50	Thailand	[30]
*O. marmorata*	46	4sm + 42a	50	Indonesia	[33]
*O. marmorata*	46	2m + 2sm+ 42a	50	Thailand	[34]
